# Comparative Physiological and Transcriptomic Analyses Reveal Mechanisms of Improved Osmotic Stress Tolerance in Annual Ryegrass by Exogenous Chitosan

**DOI:** 10.3390/genes10110853

**Published:** 2019-10-28

**Authors:** Junming Zhao, Ling Pan, Man Zhou, Zhongfu Yang, Yu Meng, Xinquan Zhang

**Affiliations:** 1 Department of Grassland Science, Sichuan Agricultural University, Chengdu 611130, Sichuan, China; junmingzhao163@163.com (J.Z.); panling1199@126.com (L.P.); yangzf211@163.com (Z.Y.); 2 College of Natural, Applied and Health Sciences, Wenzhou Kean University, Wenzhou 325060, Zhejiang, China; Man.Zhou.Research@gmail.com

**Keywords:** *Lolium multiflorum* Lam., antioxidant enzymes, osmotic stress, transcriptome, exogenous chitosan, physiological and photosynthetic characterizes

## Abstract

Water deficit adversely affects the growth and productivity of annual ryegrass (*Lolium multiflorum* Lam.). The exogenous application of chitosan (CTS) has gained extensive interests due to its effect on improving drought resistance. This research aimed to determine the role of exogenous CTS on annual ryegrass in response to water stress. Here, we investigated the impact of exogenous CTS on the physiological responses and transcriptome changes of annual ryegrass variety “Tetragold” under osmotic stress induced by exposing them to 20% polyethylene glycol (PEG)-6000. Our experimental results demonstrated that 50 mg/L exogenous CTS had the optimal effect on promoting seed germination under osmotic stress. Pre-treatment of annual ryegrass seedlings with 500 mg/L CTS solution reduced the level of electrolyte leakage (EL) as well as the contents of malondialdehyde (MDA) and proline and enhanced the activities of superoxide dismutase (SOD), catalase (CAT), peroxidase (POD), and ascorbic acid peroxidase (APX) under osmotic stress. In addition, CTS increased soluble sugars and chlorophyll (Chl) content, net photosynthetic rate (A), stomatal conductance (gs), water use efficiency (WUE), and transpiration rate (E) in annual ryegrass seedlings in response to three and six days of osmotic stress. Transcriptome analysis further provided a comprehensive understanding of underlying molecular mechanisms of CTS impact. To be more specific, in contrast of non-treated seedlings, the distinct changes of gene expressions of CTS-treated seedlings were shown to be tightly related to carbon metabolism, photosynthesis, and plant hormone. Altogether, exogenous CTS could elicit drought-related genes in annual ryegrass, leading to resistance to osmotic stress via producing antioxidant enzymes and maintaining intact cell membranes and photosynthetic rates. This robust evidence supports the potential of the application of exogenous CTS, which will be helpful for determining the suitability and productivity of agricultural crops.

## 1. Introduction

Chitosan (CTS) is a natural, safe, and economical carbohydrate polymer produced by deacetylation of nontoxic and bio-functional chitin from shellfish such as *Brachyura*, *Caridea* and *Procambarus clarkia*. During the past several decades, CTS has been proven to improve plant production and induce abiotic stress tolerance as a plant growth regulator [1]. For example, Guan et al. reported that maize seeds priming with CTS had better germination and growth under cold stress [2]. Pongprayoon et al. found that CTS clearly activated osmotic stress defense in rice [3]. Bittelli et al. suggested that CTS could increase water use efficiency in pepper in response to drought stress [4]. These findings elaborated the effects of exogenous CTS on the alterations of physiological response and gene expression under stress [1,5]. In plants under stress conditions, changes in antioxidant enzymes such as superoxide dismutase (SOD) and catalase (CAT) activities, electrolyte leakage (EL), relative water content (RWC), malondialdehyde (MDA) concentration, H_2_O_2_, and $O_{2}^{-}\cdot$ were evaluated in response to CTS treatment [6,7,8]. Indeed, CTS has been shown to affect drought-related genes in soybean [9] and defense-related genes in rice [10] on transcriptional level. However, few studies have taken into consideration of the critical parameters involved in CTS application, such as CTS concentration, the duration of treatment, and the developmental stages of the plants.

Annual ryegrass (*Lolium multiflorum* Lam.), an economically and agriculturally important cool season grass species, is broadly grown throughout the world for its high productivity and forage value [11]. Unfortunately, just like other crops, annual ryegrass has been suffering from prolonged water deficit mainly caused by global climate change, which thus severely reduced its potential yields in the natural pastures of semi-arid condition [12,13]. Hence, engineering annual ryegrass with improved resistance to water stress is in urgent need of full implementation. Recently, the exogenous application of CTS has gained extensive interests due to its effect on enhancing drought tolerance. If exogenous CTS also acts as a water stress enhancer for annual ryegrass, the underlying molecular mechanism yet remains unknown.

The aim of this study was to unravel the yet-unknown role of exogenous CTS on annual ryegrass in response to water deficit. Polyethylene glycol (PEG) 6000–induced osmotic stress can result in water deficit without causing any direct physiological damage in plants [14]. The alterations of physiological traits, photosynthetic traits, and transcriptome of CTS pre-treated and mock-treated ryegrass seedlings were examined under the PEG 6000–induced osmotic stress to address three main questions as following: (1) which levels of exogenous CTS are optimal to improve resistance to osmotic stress in annual ryegrass, (2) how does the exogenous CTS prevent annual ryegrass seedlings from osmotic stress, and (3) which are the CTS-induced biological processes and corresponding osmotic stress-responsive genes functioning to improve the resistance of annual ryegrass against osmotic stress?

## 2. Materials and Methods 

### 2.1. Plant Materials and Chitosan Treatments

The annual ryegrass cultivar “Tetragold” was provided by Barenbrug Co. (Beijing, China). The seeds were sterilized in 20% NaClO for 5 min, and rinsed five times with distilled water, and then 2.5 g of seeds was sown in trays (30 cm × 16 cm × 10 cm) with 1/2 Hoagland’s solution in growth room. The environmental conditions were as follows: 25 °C (day) and 20 °C (night), and photosynthetically active radiation 300 μmol m^−2^s^−1^. For the CTS treatment, when the plants had 3–4 leaves, roots of annual ryegrass were pre-treated in 1/2 Hoagland’s solution mixed with CTS for three days. To optimize the concentration of CTS, increasing concentrations of CTS (0, 50, 100, 250, 500, 1000, 2000 mg/L (*W*/*V*)) were chosen to determine the changes of leaf relative water content (RWC), leaf electrolyte leakage (EL), and malondialdehyde (MDA) content after four days of osmotic stress. As commonly used in previous studies to induce the osmotic stress in annual ryegrass, 20% polyethylene glycol (PEG)-6000 was selected for treatment in this study [15,16]. Mock- (0 mg/L CTS) or CTS-treated plants were then exposed to PEG or 1/2 Hoagland’s solution resulting in the following four different combinatory treatment groups: (1) CK, mock-treated plants exposed to 1/2 Hoagland’s solution; (2) CTS, CTS pre-treatment plants exposed to 1/2 Hoagland’s solution; (3) PEG, mock-treated plants exposed to 1/2 Hoagland’s solution containing 20% PEG-6000; and (4) PEG + CTS, CTS pre-treatment plants exposed to 1/2 Hoagland’s solution containing PEG-6000. Leaf samples were collected after 0, 3, and 6 days of osmotic stress, respectively. For each treatment group, the physiological parameters were measured, and qRT-PCR analyses were further performed in three independent replicates.

Our preliminary experimental results showed that the germination of annual ryegrass seeds was inhibited under 400 mg/L (*W*/*V*) CTS. To characterize the seed germination, the sterilized seeds were soaked in lower concentration [0, 10, 20, 30, 40, 50, 80, 100, and 200 mg/L (W/V)] CTS for 24 h at 25 °C, respectively. Each treatment had six independent replicates (50 seeds per replicate). The soaked seeds experienced germination in 9 cm diameter petri dishes cushioned with two pieces of filter paper treated with 3 ml of 20% (*W*/*V*) PEG-6000. The petri dishes were then placed in a growth chamber where the day/night temperature was set to 25 °C and 20 °C, photosynthetically active radiation 300 μmol m^−2^s^−1^ and 75% relative humidity. Seeds were sampled at four and seven days after germination for germination vigor and germination percentage measurements, respectively. Another batch was sampled at seven days after germination for determinations of shoot length, root length, and fresh weight.

### 2.2. Seedling Physiological Analysis

The RWC in leaf was measured following the protocol of Barrs and Weatherley [17]. Upon the removal of leaves from the plant, the fresh weight (FW) of leaves was determined immediately. After 12 h submersion of the leaves in deionized water at 4 °C, turgid weight (TW) was measured once the turgid leaves were blotted dry. The dry weight (DW) of leaves was then obtained following incubation at 80 °C for no less than 72 h. The formula RWC (%) = [(FW − DW)/(TW − DW)] × 100% was applied to determine the Leaf RWC. Leaf chlorophyll (Chl) concentration was determined by assessing the absorbance at 645 nm and 663 nm. Following the 72 h immersion of leaves (0.1 g) in 95% ethanol (10 mL) without the light, a spectrophotometer was used to assay the Chl content in the leaf extract.

Based on the protocol by Bates et al. [18], free proline was quantified. Upon immersing the leaves (0.1 g) in 3% sulfosalicylic acid (10 mL), the leaf tissues were then boiled in water for 20 min. Once the temperature drops to room temperature, the solution mixed with leaf extract (1 mL), glacial acetic acid (1 mL) and 2.5% ninhydrin test reaction reagent (1.5 mL) was heated in boiling water for 40 min. The ice bath incubation terminated the reaction which was then added in 2.5 mL toluene for the analytical separation. After 1 min vigorously mixing the reaction, the phases were separated, and the proline content was determined by measuring the absorbance of chromophore-containing toluene at 520 nm.

Leaf electrolyte leakage (EL) was gauged according to the method of Blum and Ebercon [19]. After washing the leaves (0.1 g) with deionized water three times, the leaves were immersed and shaken in deionized water (30 mL) for 24 h. The initial conductivity (Ci) was determined by the conductivity meter and applied to express the solution conductance. Leaves were subjected to autoclave at 120 °C for 20 min and cooled down to room temperature. The maximum conductance (Cmax) was then assayed for the tissue conductivity. The percentage of Ci/Cmax was used to express EL.

By applying the sulfanilamide method [20], the formation rate of $O_{2}^{-}\cdot$ was determined. The leaf extracts were made by mixing 0.1 g grinding leaves with 1.5 mL 65 mM PBS (pH 7.8). Upon centrifugation at 10,000 rpm for 30 min at 4 °C, the supernatant was separated for further use in the reaction. The supernatant (0.5 mL) was incubated at 25 °C water bath in the mixture with 0.5 mL PBS and 0.1 mL 10 mM hydrochloride for 20 min. Then, the reaction mixture was incubated for additional 20 min at 25 °C upon the addition of 1 mL 58 mM sulfanilamide plus 1 mL 7 mM a-naphthylamine. Finally, the absorbance of reaction being extracted via 2 mL chloroform was determined at 530 nm. H_2_O_2_ was assayed using the potassium iodide method [21]. Also, 0.1 g of leaf tissue homogenate in 5 mL 0.1% TCA was centrifuged for 20 min at 12,000 rpm. The reaction was conducted by mixing 0.5 mL leaf supernatant with 0.5 mL 10 mM potassium phosphate and 1 mL 1 M KI. The results were recorded by measuring the absorbance at 390 nm.

MDA content was examined following the protocol by Heath and Packer [22]. The leaves weighing 0.2 g were homogenized in solution of 2 mL of 20 % (*W*/*V*) trichloroacetic acid on ice. The supernatant was collected following centrifugation of tissue homogenates at 12,000 rpm for 10 min. Upon the addition of 0.5 mL supernatant along with 1 mL reaction solution (20% trichloroacetic acid and 0.5% thiobarbituric acid) into the pellet, the reaction was heated for 10 min at 95 °C and then placed in cold water to cool it down. Following the centrifugation of the reaction at 8000 rpm for 10 min, the absorbance of the reaction was assayed at 532 and 600 nm.

To determine the antioxidant activities, 0.3 g of fresh leaves were collected per tray on each sampling day in a random manner, subjected to “snap freezing” in liquid nitrogen, and later stored at −80 °C for future analyses. For extraction, the sample was ground on ice with 4 ml of 50 mM phosphate buffer (pH 7.8). The homogenate was centrifuged at 12,000 g for 20 min at 4 °C. The supernatant was used for assays of antioxidant enzyme activity. The ascorbic acid peroxidase (APX) activities were assayed based on the protocol of Nakano and Asada [23]. Here, 0.05 mL of supernatant was mixed with 1.5 mL reaction reagent containing 10 mM ascorbic acid, 0.003 mM EDTA, 5 mM H_2_O_2_, and 100 mM PBS (pH 5.8). The absorbance of reaction was determined at 290 nm every 10 s for 1 min. The peroxidase (POD) and catalase (CAT) activities were measured folloing the method of Maehly and Chance [24]. Here, 0.05 mL enzyme extract was mixed with 1.5 ml reaction reagent either composed of 0.05 mL of 0.75% H_2_O_2_, 0.5 mL of 0.25% guaiacol solution, and 0.995 mL of 100 mM PBS, pH 5.0 for POD assay or 0.5 mL of 45 mM H_2_O_2_ and 1 mL of 50 mM PBS, pH 7.0 for CAT assay. Results of POD or CAT assays were recorded as the absorbance measured every 10 s for 1 min at 470 or 240 nm, respectively. The SOD activities were examined using a total superoxide dismutase (SOD) Assay Kit (S0102; Haimen Beyotime, Haimen, China). All these relative enzyme activities were expressed as fold change. Protein concentration (mg·g^−1^ DW (dry weight)) was analyzed using bovine serum albumin (BSA) as a standard according to Bradford [25]. 

### 2.3. Measurement of Photosynthetic Characteristics 

Six seedling leaves from three trays were measured for the photosynthetic parameters by using a narrow leaf chamber connected to a CIRAS-3 (PP Systems, Amesbury, MA, US) [26]. In this study, 400 µmol mol^−1^ of carbon dioxide was constantly retained, and 800 µmol m^−2^s^−1^ of light intensity was maintained with the light-emitting diode light sources placed in the leaf chamber.

### 2.4. RNA Extraction and Transcriptome Sequencing Analysis

Three independent replicates of the mock-treated and 3 d of CTS pre-treatment seedlings were collected for RNA-seq analysis. Total RNA was isolated from plant leaf with Plant Total RNA Kit (Qiagen, Germantown, MD, USA). The RNA samples were qualified and quantified by running 1% agarose gels and Qubit^®^ 2.0 Flurometer (Life Technologies, Foster, CA, USA). RNA-seq libraries were constructed by using NEBNext^®^ Ultra™ RNA Library Prep Kit for Illumina^®^ (NEB, Ipswich, MA, USA) according to the manufacture’s protocol. Followed by the manufacture’s protocol, a cBot Cluster Generation System was used to perform the clustering of the index-coded samples. An Illumina (San Diego, CA, USA) Hi-seq platform was used to sequence the library, and the paired-end reads were produced. The software Trinity was used to assemble the annual ryegrass transcriptome based on left.fq and right.fq [27], and all the parameters used the default settings except that the parameter “min kmer cov” was set to 2. Seven public databases—cluster of orthologous groups of proteins database (COG), kyoto encyclopaedia of genes and genomes database (KEGG), gene ontology database (GO), non-redundant protein database (NR), non-redundant nucleotide database (NT), Swiss-Prot protein database (Swiss-Prot), and protein family database (Pfam)—were used as references to annotate the gene functions. A user-friendly software package RSEM (RNA-Seq by Expectation Maximization) (http://deweylab.biostat.wisc.edu/rsem) was used to quantify transcript abundances [28]. Differentially expressed genes (DEGs) analysis and comparisons between two conditions were carried out according to the DESeq R package (v1.10.1) (http://bioconductor.org/biocLite.R). Goseq R packages (http://bioinf.wehi.edu.au/software/goseq/) [29] and the software KOBAS (http://genome.cbi.pku.edu.cn/download.html) [30] were used to detect the significance of pathway enrichment of the DEGs in GO and KEGG analysis, respectively. 

### 2.5. Isolation of Plant RNA and Expression Analysis

Total RNA was isolated from plant leaf with Plant Total RNA Kit (Qiagen, Germantown, MD, USA). First strand cDNAs were generated using PrimeScript^TM^ RT reagent Kit (TaKaRa, Dalian, China) along with 1 µg of total RNA as the template and 50 pmol Oligo dT primer in 20 µL, following the manufacturer’s instruction manual. Reactions were performed as follows: 42 °C for 2 min, 37 °C for 15 min, and 85 °C for 5 s. Sosofast Supermix mixture (Bio-Rad, Hercules, CA, USA) was used on the Bio-Rad CFX96 detection system by the manufacturer’s instructions. The qRT-PCR program was set as follows: 30 s denaturation at 95 °C, followed by 42 cycles of 95 °C for 5 s and 58 °C for 10 s. Three technical replicates were calculated for each biological sample. The *LmActin* gene was used as the internal reference in annual ryegrass for real-time PCR according to Pan et al. [11]. The relative expression levels were normalized to that of *LmActin* and presented using 2^−ΔΔCt^ method [31]. All primer pairs used in the study were listed in Table S1.

### 2.6. Statistical Analysis 

Two-way ANOVA statistical analysis was conducted on the data. Fisher’s protected LSD at the probability of 0.05 (SPSS version 19, SPSS Inc., Chicago, IL, USA) was applied to separate the means of different treatments. The format of means ± standard deviation (SD) was used to present the data in all figures.

## 3. Results

### 3.1. Effects of CTS on Seed Germination under Osmotic Stress

As shown in Table 1, the germination of annual ryegrass seeds was significantly affected by 20% PEG-6000. The germination vigor of the “Tetragold” cultivar was severely inhibited, and the germination percentage of seeds treated by PEG-6000 was below 30%. However, pre-treatment with CTS at the following concentrations—30, 40, 50, 80, and 100 mg/L—significantly improved the germination vigor as well as germination rate in the “Tetragold” cultivar. Furthermore, exogenous application of CTS apparently stimulated the growth of roots and shoots of annual ryegrass (Table 1). Meanwhile, the fresh weight of the seedlings with CTS pre-treatments (10, 20, 30, 40, 50, 80 mg/L) to undercuts was increased by 14.8%, 16.4%, 18.4%, 23.7%, 26.5%, and 26.2% as compared to those without CTS treatment in response to the 20% PEG stress. Taken together, CTS application at the concentration of 50 mg/L displayed the optimal effect on promoting seed germination under osmotic stress (Table 1).

### 3.2. CTS Improved the Resistance to Osmotic Stress of Annual Ryegrass Seedlings

To further explore the effects of exogenous CTS pre-treatments, RWC, EL, and MDA content were measured in annual ryegrass seedlings four days post exposure to osmotic stress. There are significant differences in the EL value and MDA content among mock and different concentrations CTS pre-treatments (Table 2). After the four-day PEG treatment in mock pre-treated seedlings, the leaf RWC was reduced to 47.20%, while the leaf EL value and MDA content were increased to 31.45% and 13.22 nmol/L. CTS pre-treatment increased the leaf RWC in seedlings as compared to those with mock pre-treatment. CTS also significantly slowed down the accumulation of EL value and MDA content in the PEG-treated seedlings. However, exogenous application of CTS at concentrations higher than 500 mg/L could not further increase leaf RWC and decrease EL and MDA accumulation. Overall, the pre-treatment of ryegrass seedlings with CTS at the concentration of 500 mg/L or below improved the resistance to osmotic stress as indicated by significant increase in RWC value and reduction in the EL value and MDA content.

### 3.3. Application of CTS Positively Contributes to Osmotic Stress Tolerance by Impacting Physiological Traits

**Figure 1:**
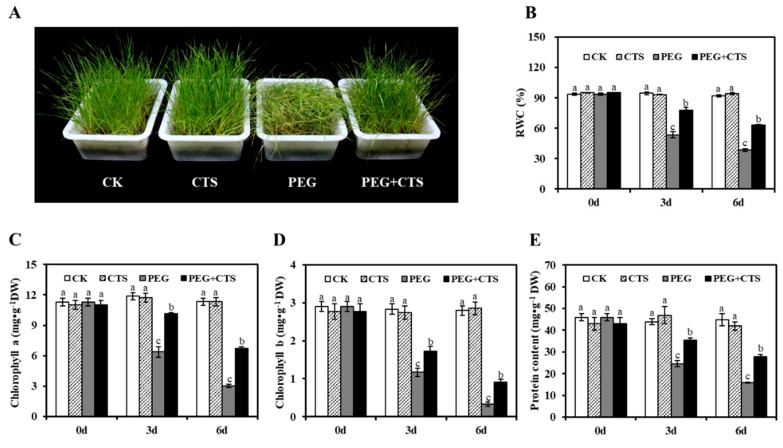
Chitoscan (CTS) pretreatment improved annual ryegrass resistance to osmotic stress tolerance. (**A**) Phenotype of annual ryegrass after polyethylene glycol (PEG) treatment with or without CTS. Effect of CTS on changes of annual ryegrass relative water content (**B**), chlorophyll content (**C**,**D**), and protein content (**E**) before and after osmotic stress treatment. Vertical bars represent mean values ± SD for each mean. Different letter indicates significant differences at given day (*P* ≤ 0.05).

The application of CTS at concentration of 500 mg/L has been demonstrated to improve the osmotic stress resistance in the most efficient manner when taking consideration of changes in RWC, EL value, and MDA content. Therefore, 500 mg/L CTS was further used to investigate whether CTS pre-treatment impacts the physiological traits of annual ryegrass during osmotic stress. Under well-irrigated conditions, exogenous CTS had no effect on RWC, Chl (a + b) content and protein content (Figure 1). However, under osmotic stress, the phenotypes of PEG + 500 mg/L CTS-treated seedlings were greener and taller than PEG-treated seedlings without CTS application (Figure 1A). The phenotypic observations are in consistence with the measurements of RWC, Chl a and Chl b contents. After three days and six days of osmotic stress treatments, the RWC, Chl a, Chl b, and protein content of PEG-treated seedlings and PEG + CTS–treated seedlings declined significantly compared to CK seedlings (*P* < 0.05). Annual ryegrass seedlings treated with CTS showed 25% higher in RWC than untreated seedlings under osmotic stress (Figure 1B). Without CTS application, 20% PEG induced a dramatic reduction in the Chl a and Chl b contents in the seedlings. In contrast, the seedlings treated with CTS displayed significant less reduction of Chl a and Chl b compared to those treated without CTS (Figure 1C,D). In addition, in response to osmotic stress, higher protein content was observed in seedlings treated with 500 mg/L CTS seedlings than those without the application of CTS (Figure 1E). These results demonstrated that the application of CTS suppressed the negative effect of PEG treatment on annual ryegrass seedlings by significantly increasing the value of RWC, Chl a, Chl b, and protein content.

**Figure 2:**
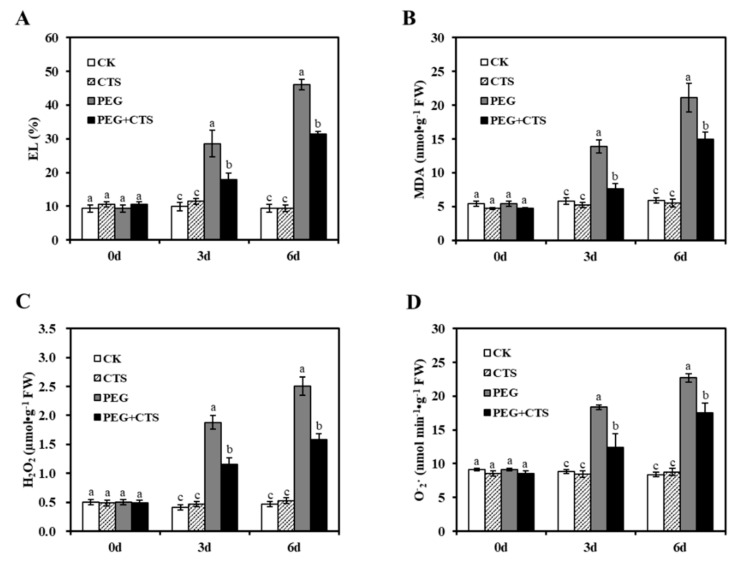
Effect of chitoscan (CTS) pre-treatment on changes of annual ryegrass EL value (**A**), MDA content (**B**), H_2_O_2_ content (**C**), and $O_{2}^{-}\cdot$ content (**D**) before and after osmotic stress treatment. Vertical bars represent mean values ± SD for each mean. Different letter indicates significant differences at given day (*P* ≤ 0.05).

**Figure 3:**
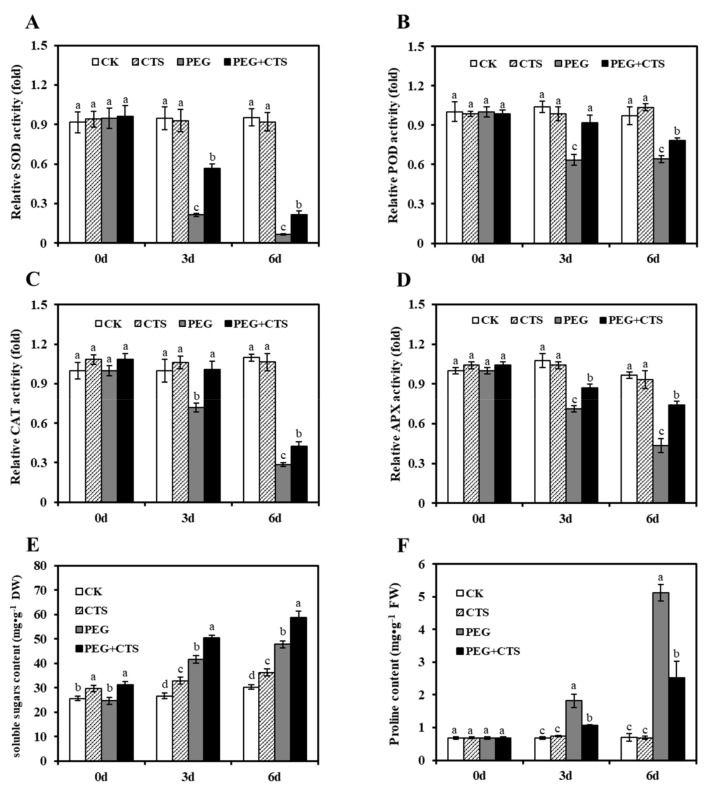
Effect of chitoscan (CTS) pre-treatment on changes of antioxidant enzymes superoxide dismutases (SOD) (**A**), POD (**B**), CAT (**C**), APX (**D**), soluble sugars (**E**), and proline (**F**) content in annual ryegrass under osmotic stress condition. Vertical bars represent mean values ± SD for each mean. Different letter indicates significant differences at given day (*P* ≤ 0.05).

To investigate the oxidative stress in annual ryegrass seedlings with and without CTS in response to osmotic stress, changes in $O_{2}^{-}\cdot$ and H_2_O_2_ content were quantified after annual ryegrass seedlings being subjected to osmotic stress for three days and six days. As shown in Figure 2, 500 mg/L CTS-treated plants reduced the accumulation of $O_{2}^{-}\cdot$ and H_2_O_2_ as compared to that in mock-treated plants when they were under three-day and six-day osmotic stressed conditions. Similarly, 500 mg/L CTS-treated plants had significantly lower MDA content and EL value under osmotic stress (Figure 2C,D). As major indicators of the stress-triggered ROS level oxidative damage, activities of antioxidant enzyme were measured in seedlings treated with PEG, CTS and PEG + CTS. Under the osmotic stress for three days, the activities of SOD, POD, CAT, and APX in PEG + CTS–treated seedlings were 19.66%, 11.24%, and 24.97% higher than those in PEG treatment, respectively (Figure 3). After 6sixdays of osmotic stress, the SOD, POD, CAT, and APX activities in the PEG + CTS–treated seedlings were 15.24%, 11.91%, and 7.62% higher than those in PEG-treated seedlings, respectively (Figure 3A–D). These data indicated that CTS could aid in reducing the stress of cell damage by decreasing the accumulation of reactive oxygen species through increasing the APX, CAT, POD, and SOD activities. During the six days of dehydration stress induced by PEG, no obvious change in the proline content of seedlings was identified with the application of CTS as compared to that of the CK group (Figure 3F). However, proline content in PEG + 500 mg/L CTS–treated plants was 0.8 and 1 times lower than that in PEG-treated seedlings under three days and six days of dehydration stress, respectively (Figure 3F), indicating that osmotic stress did cause proline accumulation in plant cells.

### 3.4. Application of CTS Mitigates the Effects of Osmotic Stress on Photosynthetic Rates

**Figure 4:**
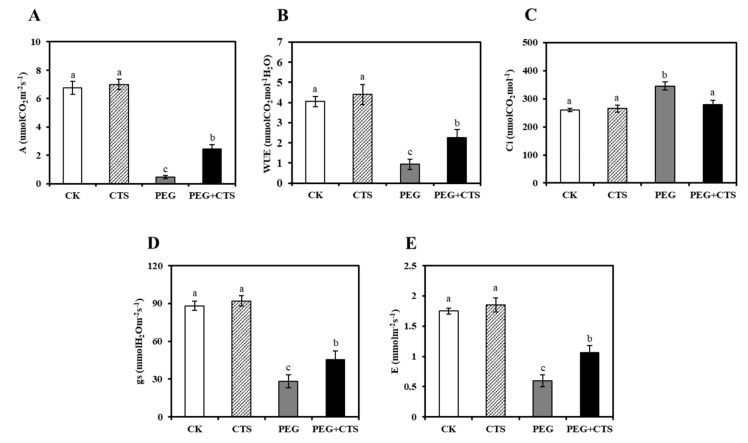
Effect of chitoscan (CTS) pre-treatment on photosynthetic characteristics net photosynthetic rate (**A**), water use efficiency (**B**), intercellular CO_2_ concentration (**C**), stomatal conductance (**D**), and transpiration rate (**E**) of annual ryegrass suffered from osmotic stress. Vertical bars represent mean values ± SD for each mean. Different letter indicates significant differences at given day (*P* ≤ 0.05).

Upon PEG treatments, the photosynthetic parameters such as net photosynthetic rate (A), stomatal conductance (gs), water use efficiency (WUE), and transpiration rate (E) tended to be declined (Figure 4). However, CTS application could restore such decline to some extent (Figure 4). Indeed, after being subjected to six days of osmotic stress, the PEG + CTS treated plants had higher values of A, gs, and WUE than those treated without CTS (Figure 4A,B,D,E). However, the intercellular CO_2_ concentration (Ci) can be maintained at the level of the CK plants (Figure 4C). This suggests that CTS can mitigate the effects of water stress on photosynthetic rates by increasing the levels of A, gs, WUE, and E.

### 3.5. The Expression of Antioxidant Enzyme-Related Genes and Proline Biosynthetic Gene P5CS1 were Affected by Exogenous CTS Application

**Figure 5:**
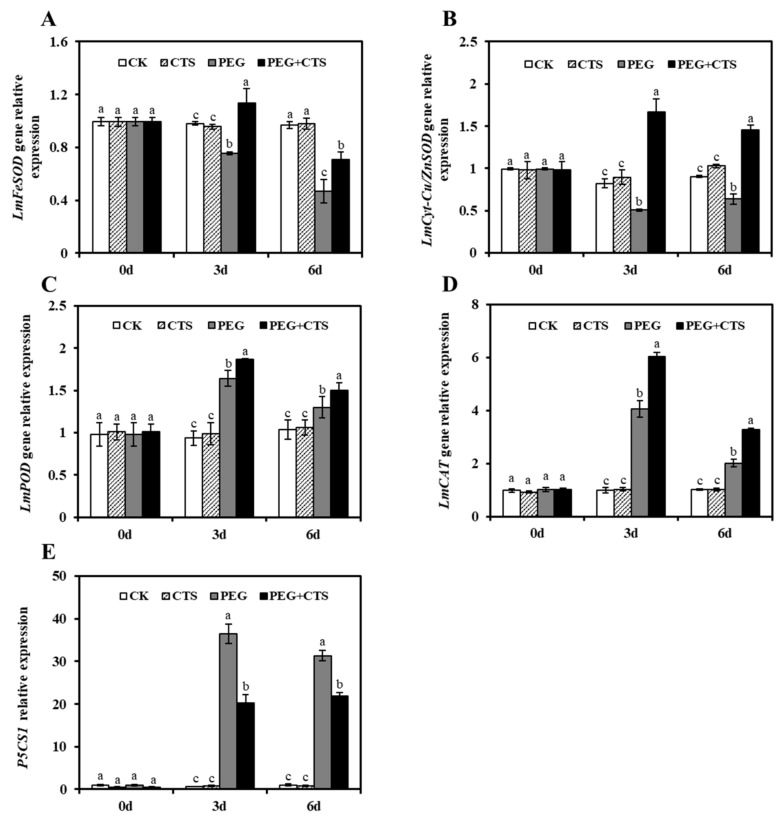
Effect of chitoscan (CTS) pre-treatment on expression level of genes *LmFeSOD* (**A**), *LmCyt-Cu/ZnSOD* (**B**), *LmPOD* (**C**), *LmCAT* (**D**), and *P5CS1* (**E**) under osmotic stress condition. The relative expression levels were normalized to that of *LmActin* and presented using the 2^−ΔΔCt^ method. Vertical bars represent mean values ± SD for each mean. Different letter indicates significant differences at given day (*P* ≤ 0.05).

Four antioxidant enzyme-related genes (*LmFeSOD*, *LmCyt-Cu/ZnSOD*, *LmPOD*, and *LmCAT*) and 1-Pyrroline-5-carboxylate synthetase 1 gene (*P5CS1*) were used to detect the alterations in gene expression profiles with 500 mg/L CTS in osmotic stress-treated annual ryegrass seedlings using qRT-PCR. Obviously, compared with PEG treatment, all four antioxidant enzyme–related genes were up-regulated under PEG 6000–induced osmotic stress. In the PEG + CTS-treated seedlings, an increase in *LmFeSOD* gene expression with three days osmotic stress along with a reduction in expression of *LmFeSOD* gene after six days of stress were observed as a result of CTS application (Figure 5A). As shown in Figure 5B, *LmCyt-Cu/ZnSOD* gene was significantly up-regulated by exogenous CTS under dehydration stress, even higher than which of the CK seedlings. Interestingly, the expression level of *LmPOD* gene in PEG + CTS–treated seedlings displayed a mixed pattern of increase then followed by decrease (Figure 5C). This is the same trend to what was observed in the *LmCAT* gene (Figure 5D). In addition, the gene expression profiles of *P5CS1* agreed with the changes in proline content in PEG + CTS-treated seedlings at three days and six days of dehydration stress (Figure 5E).

### 3.6. RNA-Seq and Data Analysis

Since exogenous CTS application for three days before being exposed to 20% PEG improved resistance to osmotic stress, which might be due to the alterations of expression levels of drought-related genes in annual ryegrass, the mock-treated and 3 d of pre-treatment seedlings were used for RNA-seq analysis to display the effects of CTS on a global scale in transcriptional level. Six RNA-libraries were sequenced, and the raw data were deposited at NCBI (accession number: PRJNA559428). After removing the low-quality reads, clean reads were generated leading to a total of 445,458 unigenes assembled with an average length of 508 nt (Figure S1A, Table 3). Seven public databases—gene ontology database (GO), non-redundant protein database (NR), non-redundant nucleotide database (NT), Kyoto Encyclopedia of Genes and Genomes (KEGG), Swiss-Prot protein database (Swiss-Prot), protein family database (Pfam), and euKaryotic Ortholog Groups (KOG)—were used as references to annotate the unigenes. Among the annotated unigenes, 165,701, 148,607, 48,144, 87,446, 115,140, 117,461, and 23,513 unigenes could be annotated to NR, NT, KO, Swissprot, Pfam, GO and KOG databases, accounting for 37.20%, 33.36%, 10.81%, 19.63%, 25.85%, 26.37%, and 5.27%, respectively (Figure S1B). Regarding the analysis, the standard of deterring differentially expressed genes (DEGs) was set to a fold-change of two or more, and false discovery rate (FDR) < 0.05. CTS application resulted in 1716 DEGs, with 1132 up-regulated and 584 down-regulated (Figure S1C). To verify the RNA-seq results, 12 genes were randomly selected for qRT-PCR analysis. Our results demonstrated the RNA-seq data is in good consistence with the qRT-PCR (Figure S2).

**Figure 6:**
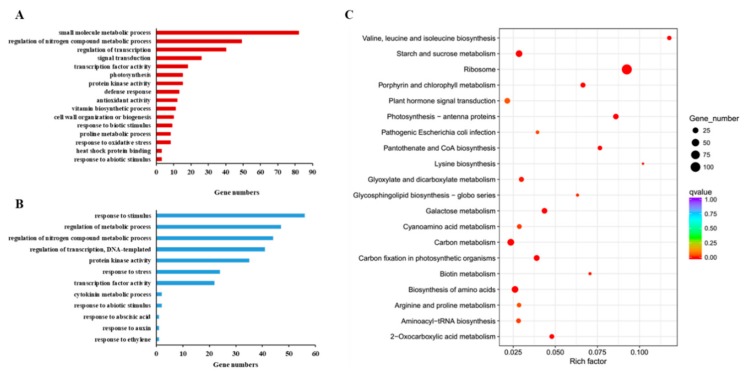
Gene ontology database (GO) and kyoto encyclopaedia of genes and genomes database (KEGG) classifications of differentially expressed genes (DEGs). (**A**) GO analysis of the upregulated genes in CTS vs. mock. (**B**) GO analysis of the downregulated genes in CTS vs. mock. (**C**) KEGG classifications of all genes between the without and with 3 d of CTS pre-treatment in annual ryegrass plants.

A Gene Ontology (GO) analysis was performed to unravel gene expression induced by CTS pre-treatment in annual ryegrass. A number of DEGs that interact directly with several pivotal processes leading plants to resistance to osmotic stress were identified (Figure 6). Some up-regulated genes were found in terms of “signal transduction,” “antioxidant activity,” and “response to oxidative stress” (Figure 6A). The down-regulated DEGs of “transcription factor activity,” “cytokinin metabolic process,” and “response to abiotic acid” were also found (Figure 6B). A KEGG pathway enrichment analysis was conducted to reveal the metabolic pathways in which the number of drought-related DEGs is involved (Figure 6C). The identified DEGs significantly enriched in the pathways were relevant to “signal transcription,” “carbon metabolism,” and “regulation of plant hormones.” The number of DGEs was also used to evaluate the importance of these metabolic processes, indicating that genes related to “carbon metabolism,” “biosynthesis of amino acids,” and “starch and sucrose metabolism” are dramatically affected by CTS pre-treatment during osmotic stress.

**Figure 7:**
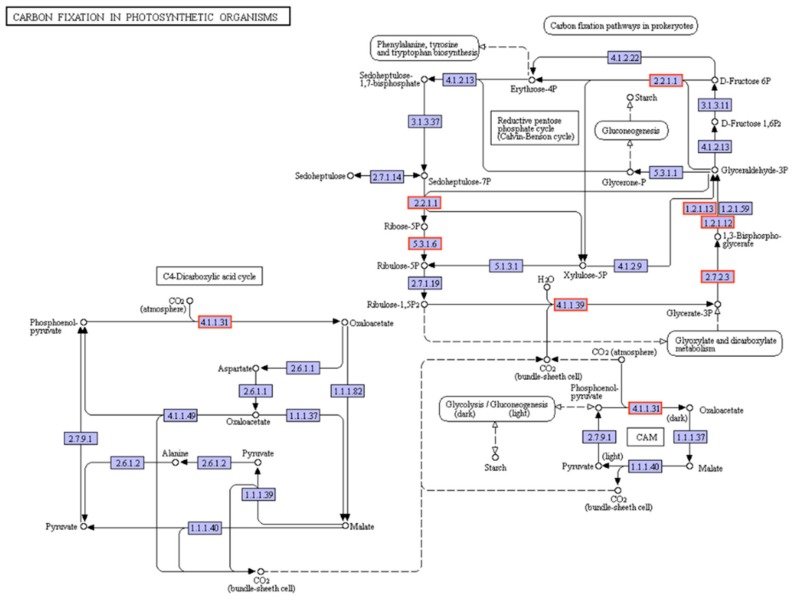
Effect of CTS on DEGs of carbon fixation in photosynthetic organisms in leaves of annual ryegrass. Definition of genes encoding enzymes: (1) 2.2.1.1, transketolase (TKT); (2) 1.2.1.13, glyceraldehyde-3-phosphate dehydrogenase (GAPDH/GAPA); (3) 5.3.1.6, ribose-5-phosphate isomerase 2 (RPI2); (4) 4.1.1.39, ribulose bisphosphate carboxylase small chain (rbcS); (5) 2.7.2.3, phosphoglycerate kinase (PGK); (6) 4.1.1.31, phosphoenolpyruvate carboxylase 1(PPC1). Red means an up-regulation.

**Figure 8:**
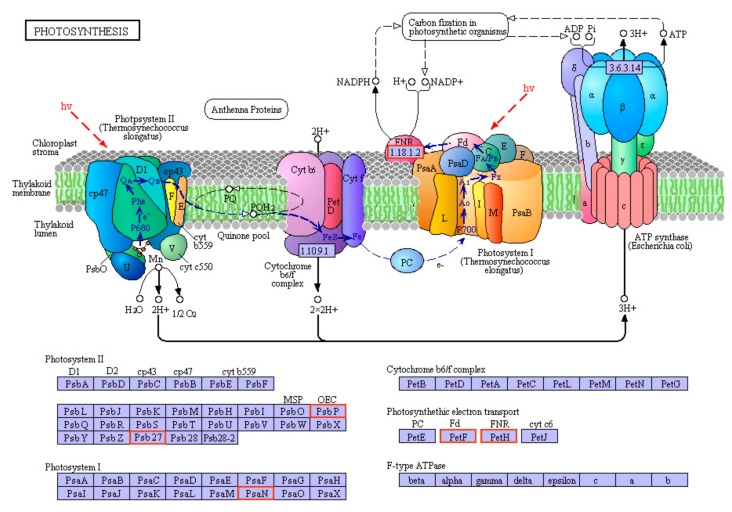
The metabolic pathway involved in photosynthesis. Definition of genes encoding proteins: (1) PsbP, photosystem II oxygen-evolving enhancer protein 2; (2) Psb27, photosystem II Psb27 protein; (3) PsaN, photosystem I subunit PsaN; (4) PetF, ferredoxin; (5) PetH, ferredoxin-NADP^+^ reductase. Red boxes indicated an up-regulation.

The osmotic pressure was relieved in exogenous CTS-pretreated annual ryegrass plants by accumulating more soluble sugars than mock-pretreated plants in response to osmotic stresses (Figure 3E). The carbon metabolism–related genes such as transketolase (TKT) (EC:2.2.1.1), glyceraldehyde-3-phosphate dehydrogenase (GAPDH/GAPA) (EC:1.2.1.13), ribose-5-phosphate isomerase 2 (RPI2) (EC:5.3.1.6), ribulose bisphosphate carboxylase small chain (rbcS) (EC:4.1.1.39), phosphoglycerate kinase (PGK) (EC:2.7.2.3), and phosphoenolpyruvate carboxylase 1(PPC1) (EC:4.1.1.31) showed up-regulated expression in CTS pre-treated annual ryegrass (Figure 7; Table S2). In addition, the expression levels of genes encoding photosynthesis related proteins (PsbP, Psb27, PsaN, PetF, and PetH) were increased (Figure 8; Table S2), suggesting that exogenous CTS application might positively modulate the accumulations of photosynthesis-related genes, which in turn increase plant resistance to osmotic stress.

Our transcriptomic analysis demonstrated that significant expression changes occurred in large numbers of hormone related genes such as abscisic acid (ABA), salicylic acid (SA), auxin (IAA), gibberellin (Gi), cytokinin (KT), and brassinolide (BR). For example, the genes involved in BR, IAA, and Gi biosynthesis and signaling pathways were significantly down-regulated, while the genes mainly involved in SA and KT pathways were significantly up-regulated (Table S2).

## 4. Discussion

Plants are highly sensitive to drought stress and require a relatively large amount of water for continued growth, especially in seed germination and seedling growth [32]. Plants may respond to CTS at a series of concentrations in different manners at various stages of growth and development [10,33]. In order to test this hypothesis, we applied different concentrations of CTS at seed germination and seedling stages of annual ryegrass to investigate the effects of CTS under water deficit conditions. We observed that no further positive effects on germination vigor, germination percentage, RWC, EL value, and MDA content were seen with higher CTS concentrations under osmotic stress. On the contrary, the lower CTS concentrations were shown to be more effective to induce osmotic stress defense. Moreover, 50 mg/L CTS application significantly facilitated the growth of roots and shoots at the post-germination stage, suggesting that CTS positively regulate the growth of annual ryegrass challenged with osmotic stress.

Water deficit limits photosynthesis, alters cell homeostasis, and causes an enhanced germination of ROS [34,35]. To meet the challenge caused by ROS, a delicate enzymatic antioxidant system involving ascorbic acid peroxidase (APX), catalase (CAT), peroxidase (POD), and superoxide dismutases (SOD) evolved to catalyze the reaction to change $O_{2}^{-}\cdot$ into H_2_O_2_ and detoxify the H_2_O_2_ product [36,37]. Our study supported the previous studies in demonstrating that CTS can enhance resistance to oxidative stress in plants [38,39]. We found that exogenous CTS treatment resulted in significant decrease in $O_{2}^{-}\cdot$ and H_2_O_2_ after three days of osmotic stress, and six days of osmotic stress showed even more reduction. However, there was no clear difference between CK and CTS treatments. This might be due to that CTS could improve the accumulation of protective enzymes (SOD, CAT, POD, and APX) in annual ryegrass seedlings under osmotic stress, indicating that CTS is able to scavenge superoxide anion as well as antioxidant during drought [40]. In agreement with this assumption, elevated activities of SOD, APX, and POD resulted from the CTS treatment were detected in previous studies [41]. It is worth noting that, in contrast with PEG treatment, the application of exogenous CTS under the osmotic stress remarkably increased the activity of antioxidants to reduce the damage caused by the accumulation of $O_{2}^{-}\cdot$ and H_2_O_2_. It suggests that the pre-treatment of CTS partially alleviates the formation of ROS by activating a variety of antioxidants in plants when they are exposed to unfavorable conditions. In addition to activity of the protective enzymes, chlorophyll, and three other molecules including free proline, protein, and soluble sugar functioning in osmotic regulation are considered as direct indicators to evaluate the water pressure induced damage in plants [42]. Compared to pre-treatment of PEG alone, results demonstrated that levels of all of these indicators were elevated during the treatment course in response to the mixture of CTS and PEG pre-treatment, except that proline content was found to be declined. It suggests that the addition of CTS is capable of strengthening the osmosis regulation as indicated by the increase in protein content and soluble sugar. Furthermore, as one of the prominent osmolyte, proline has been extensively shown to be overproduced in stress condition to aid in stress tolerance by maintaining the osmotic balance [43]. Reduction of proline content post PEG and CTS pre-treatment implies that CTS has a role in enhancing plant resistance to osmotic stress.

In general, EL levels and MDA concentrations served to indicate membrane intactness and stability [44]. Our study demonstrated that osmotic stress would markedly increase EL and MDA content in annual ryegrass seedlings, but exogenous CTS treatments at any concentration level could mitigate these effects, suggesting that CTS could restrain damages caused by osmotic stress and maintain the membrane integrity and stability. Leaf RWC is commonly proposed as a reliable indicator of leaf water status [45]. CTS treatment not only balances between water supply and leaf transpiration rate as supported by RWC data but also restores the chlorophyll synthesis abilities under osmotic stress [46]. In annual ryegrass, CTS took part in processes of maintaining normal level of RWC, chlorophyll and protein content. As dehydration stress progresses, biochemical constraints may directly limit photosynthesis [47]. CTS has been shown to enhance drought tolerance by increasing the efficiency of water use in pepper [4]. In the current study, the exogenous CTS treatment applied to seedlings resulted in four times increase in net photosynthetic rate than untreated plants at six days of osmotic stress. Though osmotic stress adversely affects photosynthesis in this experiment, CTS treatment slightly increased the net photosynthetic rate, water use efficiency, and stomatal conductance of annual ryegrass under osmotic stress. In the present study, a multitude of genes encoding the proteins functioning in photosystem II reaction center subunit such as PsbP, Psb27, PsaN, PetF, and PetH were up-regulated in annual ryegrass seedlings with CTS pre-treatment under osmotic stress conditions. Given that the multifunction of the specific proteins in photosynthesis that are proven [48], CTS pre-treatment might be able to promote plants to generate the proteins for the purpose of eliminating the unfavorable effects on the process of photosynthesis caused by osmotic stress. This finding elucidates the part of reason why CTS pre-treated plants can maintain to some extent higher photosynthetic rate than that of untreated plants exposed to water deficit.

Six proteins involved in carbon metabolism were identified. Among them, a transketolase encoded by the *TKT* gene is a pivotal enzyme associated with both the pentose phosphate pathway and the calvin cycle of photosynthesis. As a central enzyme in glycolysis, it has been widely proved that GAPDH (EC:1.2.1.13) was involved in several abiotic stress response and improved plant tolerance against the stressful conditions [49,50]. A high level of GAPDH (EC:1.2.1.13) was observed under osmotic stress, which might be a beneficial consequence of exogenous CTS treatment on seedlings of annual ryegrass. In addition, PGK (EC2.7.2.3) is one of key enzymes in the glycolytic pathway. Several studies has been focusing on the function of PGK (EC2.7.2.3) proteins in improving yield in plants under several abiotic stress such as salinity stress and drought stress [51,52]. In the present study, a gene that encodes PGK was induced to enhance the plant tolerance. Ribose-5-phosphate isomerase (RPI) was identified as a key enzyme of carbon metabolism. A decreased level in RPI expression was found in photosynthesis in drought-treated rice leaves [53]. Nevertheless, the up-regulated gene encoding RPI2 (EC:5.3.1.6) was observed in annual ryegrass, which contributes to energy consuming for resistance to osmotic stress when plants are exposed to CTS pre-treatment. Ribulose-1,5-bisphosphate carboxylase/oxygenase (RuBisCO) is a bifunctional enzyme in charge of the Calvin cycle [54]. Ribulose bisphosphate carboxylase small chain (rbcS) (EC:4.1.1.39) is one of the small subunits of RubisCO that widely exists in higher plants, but its function is not yet fully known [55]. According to the results of this study, rbcS encoded by a single gene and could result in higher plant resistance to osmotic stress after exogenous CTS treatment. Shi et al. reported that PPC1 (EC:4.1.1.31) in leaves plays a pivotal role in carbon and nitrogen metabolism in *Arabidopsis* [56]. Consistent with the previous research, the up-regulated gene encoding PPC1 (EC:4.1.1.31) that is involved in carbon metabolism was also observed in osmotic stress-treated annual ryegrass, indicating that exogenous CTS plays an important role by inducing PPC1-related genes in preventing plants from osmotic stress condition.

**Figure 9:**
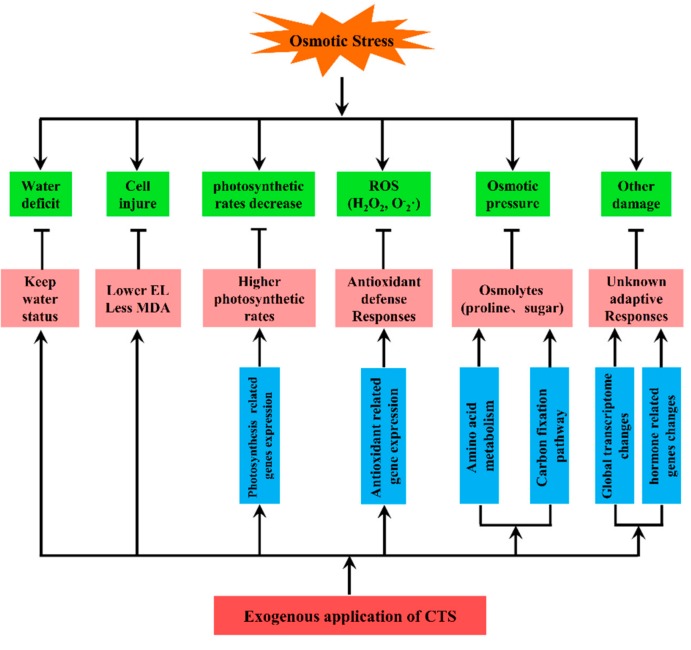
A proposed model for CTS-mediated osmotic stress responses in annual ryegrass.

Based on above-reported results, a model of CTS-mediated osmotic stress response in annual ryegrass was proposed (Figure 9). Though osmotic stress induces the ROS accumulation, osmotic pressure, and cell damage in plants, exogenous application of CTS could maintain lower MDA content and higher photosynthetic rates, change antioxidant enzyme activities, reduce proline content, and develop other unknown adaptive reactions. Meanwhile, the transcriptomic analysis revealed alterations in a variety of genes involved in photosynthesis, the carbon fixation pathway, hormone regulation, and amino acid metabolism induced by CTS pre-treatment. All these changes, in turn, relieve damage to cell integrity, ROS production, reaction to osmotic pressure, and other osmotic stress–elicited negative effects. Taken together, our study demonstrated that exogenous application of CTS could significantly improve plant performance under osmotic stress by modulating ROS accumulation, antioxidant responses, osmolyte accumulation, and expression of stress-related genes on both germination and seedling stages of annual ryegrass.

## Figures and Tables

**Table 1:** Characteristics of annual ryegrass seed germination with water or increasing concentrations of CTS pretreatment under osmotic stress. Values are mean ± SD (*n* = 10).

CTS (mg/L)	Germination Vigor (%)	Germination Percentage (%)	Shoot Length (cm)	Root Length (cm)	Seedling Fresh Weight (mg·5 Seedling^−1^)
mock	73.54 ± 3.34 a	91.22 ± 2.33 a	14.55 ± 2.38 a	9.47 ± 2.32 a	1220.27 ± 12.33 a
0	22.86 ± 2.86 de	32.57 ± 4.33 fg	1.97 ± 0.28 f	3.43 ± 0.65 f	83.03 ± 3.86 f
10	23.81 ± 4.36 de	35.42 ± 4.78 fg	2.34 ± 0.40 de	4.33 ± 0.45 d	95.30 ± 3.30 cd
20	30.47 ± 3.29 cd	37.14 ± 5.71 f	2.64 ± 0.26 cd	4.97 ± 0.78 c	96.63 ± 5.20 bc
30	32.38 ± 1.64 c	44.57 ± 3.83 e	2.89 ± 0.36 bc	5.00 ± 0.76 c	98.27 ± 6.84 bc
40	33.33 ± 5.95 c	50.28 ± 1.56 d	3.08 ± 0.54 b	5.71 ± 0.60 b	102.70 ± 6.11 bc
50	42.85 ± 5.71 b	63.42 ± 5.85 b	3.11 ± 0.35 b	5.87 ± 0.92 b	105.13 ± 6.10 b
80	37.14 ± 7.56 bc	57.71 ± 5.11 c	2.93 ± 0.25 bc	5.38 ± 0.56 bc	104.77 ± 4.19 b
100	34.29 ± 4.95 c	57.14 ± 3.49 c	2.50 ± 0.24 de	4.18 ± 0.63 d	87.50 ± 2.85 de
200	20.00 ± 2.86 e	29.52 ± 3.12 g	2.22 ± 0.45 ef	3.80 ± 0.61 de	73.25 ± 2.33 g

Different letters indicate a significant difference between each treatment at different CTS concentration.

**Table 2:** The effect of different concentrations of CTS on relative water content (RWC), leaf electrolyte leakage (EL), and malondialdehyde (MDA) content in leaves of annual ryegrass under 4 days of water stress conditions.

CTS (mg/L)	RWC (%)	EL (%)	MDA (nmol·g^−1^ FW)
mock	94.63 ± 0.83 a	5.99 ± 0.05 e	3.48 ± 0.30 e
0	47.20 ± 1.32 e	31.45 ± 0.46 a	13.22 ± 0.23 a
50	55.91 ± 1.24 d	22.06 ± 2.12 b	9.70 ± 0.20 b
100	57.22 ± 1.08 d	20.61 ± 0.72 c	9.55 ± 0.23 b
250	60.59 ± 2.43 bc	18.07 ± 0.70 d	8.64 ± 0.39 c
500	61.27 ± 0.49 b	17.66 ± 0.37 d	7.87 ± 0.19 d
1000	59.47 ± 0.32 c	21.53 ± 1.43 bc	7.93 ± 0.18 d
2000	60.65 ± 1.28 bc	21.67 ± 0.58 bc	7.83 ± 0.39 d

Values are mean ± SD (*n* = 4). Different letters indicate a significant difference between each treatment under different CTS concentrations.

**Table 3:** Summary statistics of the annual ryegrass transcriptome assemblies.

Nucleotides Length (bp)	Transcripts	Unigenes
200–500	224, 311	218, 907
500–100	149, 634	146, 798
1000–2000	63, 396	16, 843
>2000	16, 843	16, 819
Total	454, 184	445, 358
Min Length	201	201
Max Length	16, 197	16, 197
Median Length	506	508
Mean Length	691	695
N50	855	861
N90	341	343

## Supplementary Materials

Supplementary materials can be found at https://www.mdpi.com/2073-4425/10/11/853/s1. Figure S1: Length distribution and annotation of unigenes; Figure S2: Expression of the selected 12 genes inferred by RNA-seq and qRT-PCR. Error bars represent standard deviation (*n* = 3); Table S1: The primers used in qRT-PCR analysis; Table S2: List of differentially expressed genes (DEGs).
