# Supplementary material for: Comparative Physiological and Transcriptomic Analyses Reveal Mechanisms of Improved Osmotic Stress Tolerance in Annual Ryegrass by Exogenous Chitosan
Source: Genes (Basel). 2019 Oct 28;10(11):853. doi: 10.3390/genes10110853 (PMC6895815; doi:10.3390/genes10110853)
Supplement: Supplementary file 1 [file genes-10-00853-s001.pdf]

Supplementary Materials

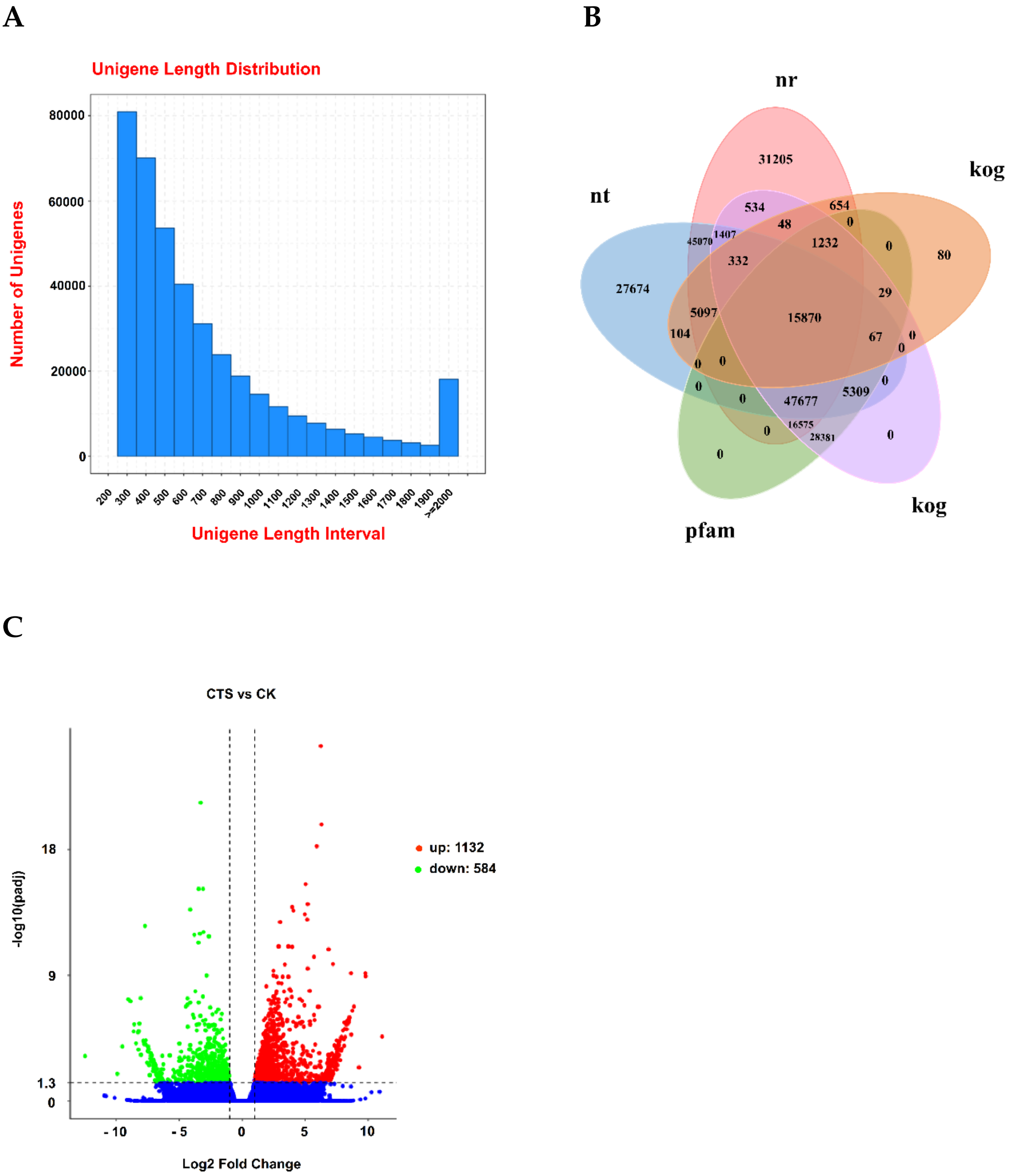

Figure S1. Length distribution and annotation of unigenes. **(A)** Unigene length distribution. The X axis represents the length of unigenes; the Y axis represents the number of unigenes. **(B)** Venn diagram of the number of unigenes annotated in 5 databases. **(C)** Volcano plot of DEGs. The X axis represents log2 transformed fold change; the Y axis represents -log10 transformed adjusted P value, the red points represent up-regulated DEGs, the green points represent down-regulated DEGs and the blue points represent non-DEGs.

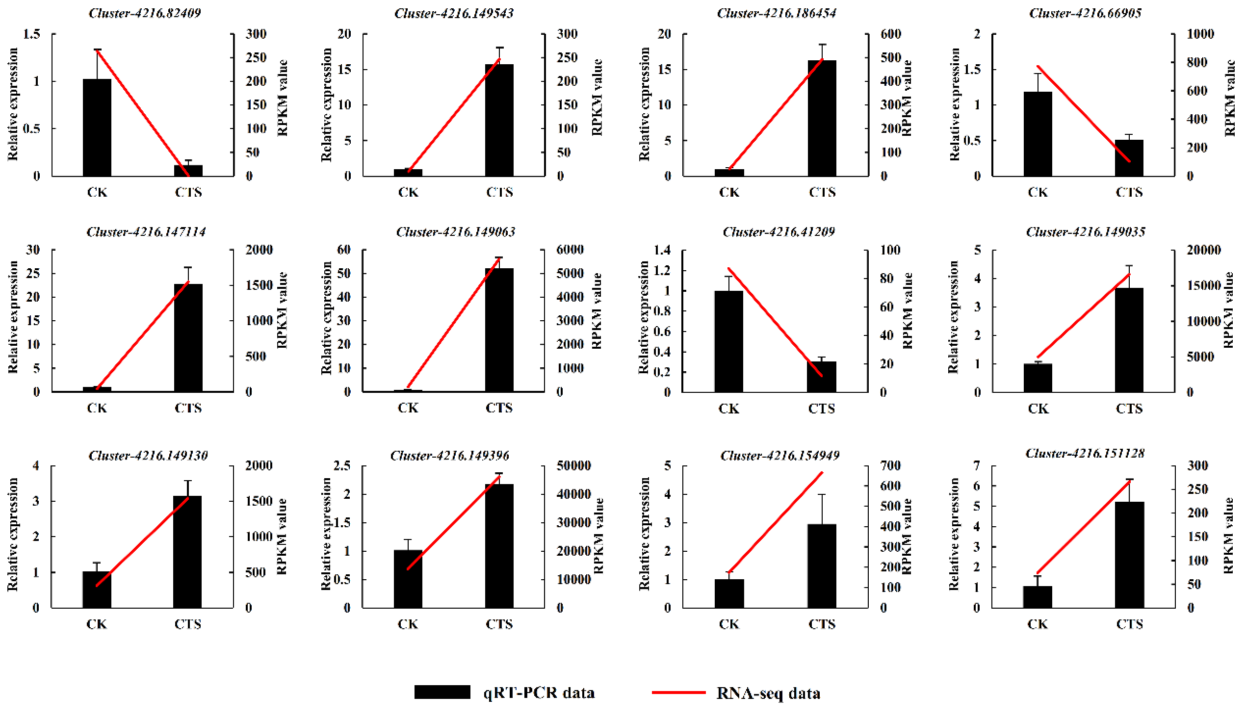

Figure S2. Expression of the selected 12 genes inferred by RNA-seq and qRT-PCR. Error bars represent standard deviation (n = 3). qRT-PCR data were obtained using $2^{-\Delta\Delta Ct}$ method, RNA-seq data represent Fragments per Kilobase Million (FPKM) value.

Table S1. The primers used in qRT-PCR analysis.

Gene	F Sequence (5' - 3')	R Sequence (5' - 3')
Cluster-4216.82409	TACCAGCCAGATTGGGAAAC	ATGTCACCTTGGAGGCTATTG
Cluster-4216.149543	CACGAGTTCTTGCTCGGATAA	GGAACCTGCGGATCTTGTT
Cluster-4216.186454	CACGAGTTCTTGCTCGGATAA	GGAACCTGCGGATCTTGTT
Cluster-4216.66905	ACATATCCACGGAGCAGTAAAT	GCGTCGAAACTTAGGTGTATTG
Cluster-4216.147114	TCACTTGCACACTGAAGAAGAT	CGTGGCGACGAGGAAAG
Cluster-4216.149063	CTACGAGGTTGGATCACGATTAC	GTGCATATATCGCCCTGTACG
Cluster-4216.41209	GAAGCTAGTAGTAATCCGTCAGC	ATGGATTTGTTCATCCTTCACTTG
Cluster-4216.149035	CGATGACAACTCCGCTTTCT	TGACGGGTCACAAACTCTTTAC
Cluster-4216.149130	ACTACTTGATCCGCTCCAAATG	AGTACCTGCCGTCGAAGTAT
Cluster-4216.149396	TGGCTTGAAGGCGATGAA	TGTAGGAGGTCAAGAAGGAGTA
Cluster-4216.154949	GAACGCCTGGTTCACCTC	CCTCAAGGGCAAGCTCAA
Cluster-4216.151128	CAACGATGGCTATGACGAGAT	AGATCCAAACCCGATTGTAGTG
LmActin	TCCTCACGCCATTCTT	TCTCCTTGATGTCCCT
LmFeSOD	CTGGTTGGGTTTGGCTTGT	TCGCCTGTCATCCTTGTAATCT
LmCyt-Cu/ZnSOD	GGCTGAGTATCCCATTT	CTGCCTTTGCTGTTCT
LmPOD	CACAGTCTGGGAAAGG	GGTCCACATAGCGTCT
LmCAT	GTGCTCAACCGCAACAT	AGCAGCAGGTAGTTCGGTC
P5CS1	ATAACCAATGCTATCCCTGAC	TCTTAGTCGTTGCCTTGA

Table S2. List of differentially expressed genes (DEGs).

	Gene ID	Putative annoation	Fold Change (Log2 FC)	Function Bin
Hormone	Cluster-4216.162893	Auxin-responsive protein IAA3	-1.3189	Auxin
	Cluster-4216.159710	Auxin-responsive protein IAA17	-2.1782	
	Cluster-4216.127879	Auxin-responsive protein IAA17	-1.2972	
	Cluster-4216.160188	Probable protein phosphatase 2C 6	-1.4591	ABA
	Cluster-4216.123385	Probable protein phosphatase 2C 6	-2.0059	
	Cluster-4216.57388	Probable protein phosphatase 2C 9	-1.8179	
	Cluster-4216.311792	Probable protein phosphatase 2C 30	-1.6566	
	Cluster-4216.57385	Probable protein phosphatase 2C 49	-1.7867	
	Cluster-4216.307484	Probable protein phosphatase 2C 68	-2.388	
	Cluster-4216.270744	ABSCISIC ACID-INSENSITIVE 5-like protein 7	-3.4758	
	Cluster-4216.140285	Transcription factor TGA1	-1.4891	Gi
	Cluster-4216.41209	Transcription factor TGA3	-2.8932	
	Cluster-4216.41201	Transcription factor TGA4	-1.9332	
	Cluster-4216.164438	Transcription factor APG	1.8308	
	Cluster-4216.166110	Pathogenesis-related protein PRB1-2	2.3803	SA
	Cluster-4216.303056	Probable serine/threonine-protein kinase	-4.0003	BR
	Cluster-4216.166723	Two-component response regulator ARR8	1.5472	KT
	Cluster-4216.157994	Two-component response regulator ARR8	1.5283	
	Cluster-4216.320437	Histidine-containing phosphotransfer protein 4	-2.1177	
Photosynthesis	Cluster-4216.66931	Ferredoxin-1	3.5141	
	Cluster-4216.152814	Ferredoxin-1	1.8225	
	Cluster-4216.66929	Ferredoxin-1	3.548	
	Cluster-4216.154693	Ferredoxin-2	1.3764	
	Cluster-4216.145950	Ferredoxin, chloroplastic	2.1005	
	Cluster-4216.152742	Ferredoxin--NADP reductase, leaf isozyme, chloroplastic	2.4224	
	Cluster-4216.144916	Oxygen-evolving enhancer protein 2, chloroplastic	5.8014	
	Cluster-4216.155340	Photosystem I reaction center subunit N, chloroplastic	1.6275	
	Cluster-4216.149748	Photosystem II repair protein PSB27-H1, chloroplastic	1.8847	
Carbon fixation in photosynthetic organisms	Cluster-4216.119189	Phosphoglycerate kinase, chloroplastic	1.5865	E.2.7.2.3
	Cluster-4216.127201	Phosphoenolpyruvate carboxylase 1	1.2861	E.4.1.1.31
	Cluster-4216.149035	Ribulose bisphosphate carboxylase small chain, chloroplastic	1.731	E.4.1.1.39
	Cluster-4216.149095	Ribulose bisphosphate carboxylase small chain PW9, chloroplastic	1.6953	E.4.1.1.39
	Cluster-4216.149121	Ribulose bisphosphate carboxylase small chain clone 512	1.7816	E.4.1.1.39
	Cluster-4216.149130	Ribulose bisphosphate carboxylase small chain PW9, chloroplastic	2.3144	E.4.1.1.39
	Cluster-4216.149139	Ribulose bisphosphate carboxylase small chain PWS4.3, chloroplastic	1.7955	E.4.1.1.39
	Cluster-4216.149140	Ribulose bisphosphate carboxylase small chain PWS4.3, chloroplastic	1.2312	E.4.1.1.39
	Cluster-4216.149192	Ribulose bisphosphate carboxylase small chain, chloroplastic	1.4394	E.4.1.1.39
	Cluster-4216.149194	Ribulose bisphosphate carboxylase small chain PWS4.3, chloroplastic	1.4753	E.4.1.1.39
	Cluster-4216.149196	Ribulose bisphosphate carboxylase small chain, chloroplastic	1.4715	E.4.1.1.39
	Cluster-4216.149236	Ribulose bisphosphate carboxylase small chain	1.6478	E.4.1.1.39
	Cluster-4216.149303	Ribulose bisphosphate carboxylase small chain PW9, chloroplastic	1.8642	E.4.1.1.39
	Cluster-4216.149342	Ribulose bisphosphate carboxylase small chain PW9	1.5069	E.4.1.1.39
	Cluster-4216.149396	Ribulose bisphosphate carboxylase small chain clone 512	1.7521	E.4.1.1.39
	Cluster-4216.149480	Ribulose bisphosphate carboxylase small chain clone 512	1.477	E.4.1.1.39
	Cluster-4216.149485	Ribulose bisphosphate carboxylase small chain PWS4.3, chloroplastic	1.3153	E.4.1.1.39
	Cluster-4216.159463	Ribulose bisphosphate carboxylase small chain clone 512	1.5728	E.4.1.1.39
	Cluster-4216.142092	Ribose-5-phosphate isomerase 2	1.5361	E.5.3.1.6
	Cluster-4216.149516	Glyceraldehyde-3-phosphate dehydrogenase A, chloroplastic	1.396	E.1.2.1.12/1.2.1.13
	Cluster-4216.149648	Glyceraldehyde-3-phosphate dehydrogenase A, chloroplastic	1.1286	E.1.2.1.12/1.2.1.13
	Cluster-4216.154949	Glyceraldehyde-3-phosphate dehydrogenase A, chloroplastic	1.9379	E.1.2.1.12/1.2.1.13

Cluster-4216.150570	Glyceraldehyde-3-phosphate dehydrogenase 3, cytosolic	1.5657	E.1.2.1.12/1.2.1.13
Cluster-4216.151128	Transketolase, chloroplastic	1.8507	E.2.2.1.1
